# The validity of the Meaning in Life in Persons with Dementia Questionnaire (MIND)

**DOI:** 10.3389/fpsyg.2025.1633401

**Published:** 2025-08-14

**Authors:** Torgeir Sørensen, Sverre Bergh, Knut Asbjørn Hestad, Ingvild Hjorth Feiring, Lars Johan Danbolt, Bjørn Lichtwarck

**Affiliations:** Innlandet Hospital Trust, Brumunddal, Norway

**Keywords:** dementia, nursing homes, meaning in life, quality of life, depression, validation, MIND questionnaire

## Abstract

**Background:**

Meaning in life is considered an underestimated asset for people’s well-being, particularly among individuals with dementia residing in nursing homes. However, knowledge on meaning in life in this target group is scarce, among other reasons because an instrument specifically developed and adapted to assess meaning in life in this population has been missing. Although existential experiences are known to affect well-being in older adults, few tools exist to assess meaning in life in individuals with dementia. This study aims to validate the newly developed Meaning in Life in Persons with Dementia Questionnaire (MIND) for use in nursing home residents.

**Methods:**

We included 116 participants with dementia from 34 nursing homes in Eastern Norway. Descriptive statistics, reliability tests, confirmative factor analysis, and multiple regression for evaluation of validity.

**Results:**

Both meaningfulness (α 0.86) and crisis of meaning (α 0.92) had acceptable internal consistency. As expected, the two constructs were also highly significant and negatively correlated (−0.59). Confirmatory factor analysis showed that all goodness of fit-values were clearly on the right side of the limits for a two-factor solution (CFI 0.999). When testing for construct validity, in multivariate linear regression analysis meaningfulness was significantly associated with higher QoL-AD scores (Standardized *β* = 0.346), while crisis of meaning showed a negative but non-significant trend. Severity of depression symptoms assessed by the Cornell Scale for Depression in Dementia (CSDD) and quality of life measured by the Quality of Life in Late-Stage Dementia scale (QUALID) were not associated with meaningfulness or crisis of meaning. Lack of significant associations may be due to low statistical power and measurement differences dependent on self-report and proxy-report.

**Conclusion:**

Despite sample limitations, our findings suggest that the MIND questionnaire is a psychometrically sound instrument for assessing meaning in life among nursing home residents with dementia. Its integration into clinical practice may support more individualized, person-centered care. Future research should explore its utility in diverse cultural and care settings, as well as its longitudinal sensitivity to change.

## Introduction

### Dementia – quality of life and depression status

Dementia is a syndrome caused by different diseases of the brain, with chronic and irreversible cognitive impairment and reduction in the ability to perform activities in daily life (WHO, 2024). In Norway, approximately 15% of people 70 years old and above have dementia (Gjøra et al., 2021). Due to demographic changes in the population, with a higher proportion of older adults, the prevalence of people with dementia in Norway is expected to more than double by 2050 (Gjøra et al., 2021). In nursing homes, more than 80% of the residents have dementia, and most residents are over 80 years old when admitted to a nursing home (Røen et al., 2017).

Older people live longer, many have good health and a good quality of life (QoL), and they participate in social life in family and society (Norwegian Government, 2017). On the other hand, older people may experience changes in their environment and loss of everyday routines and social interaction when health deteriorates. Such experiences are exacerbated by the development of dementia. This situation may be accentuated in nursing homes, as demonstrated by the finding that nursing home residents with dementia show significantly lower QoL than home-dwelling older adults with dementia (Olsen et al., 2016). At the same time, the prevalence of clinically significant depressive symptoms in this patient group ranges from 10 to 52% across Europe (Giebel et al., 2016). Further, the prospects for recovery from depression are limited (Borza et al., 2019). These factors make nursing home residents with dementia particularly vulnerable compared to community-dwelling older adults.

### The concept of meaning in life and its relations to QoL and mental health

Given the psychological vulnerability of nursing home residents with dementia, understanding existential factors such as meaning in life becomes critical. Meaning in life is considered essential in individuals’ lives, and the search for meaning is considered as a fundamental element in life (Frankl, 2004). According to Schnell (2021), meaning in life is multidimensional, consisting of the three components “meaningfulness,” “crisis of meaning,” and “sources of meaning.” Meaningfulness appears as the basic trust that life is worth living, based on an appraisal of life as “coherent, significant, oriented, and belonging.” Coherence is about making sense in life, significance emphasizes life’s inherent values, orientation focuses on having goals and objectives in life, while belonging underlines affiliation with the local and global world (Martela and Steger, 2016). Crisis of meaning is the experience of life as frustratingly empty, pointless, and lacking meaning and belonging (Schnell, 2021). It is shown that meaningfulness and crisis of meaning are two different constructs that can occur simultaneously to a greater or lesser extent appear simultaneously (Schnell, 2011). This two-dimensional solution stands in contrast to early meaning in life research with the assumption that meaningfulness and crisis of meaning represented two sides of the same continuum (Crumbaugh and Maholick, 1964). Additionally, Schnell (2021) organizes sources of meaning contributing to meaning-making into several dimensions (i.e., vertical and horizontal self-transcendence, self-actualization, order and tradition, and well-being and relatedness).

In the general population, meaningfulness has proved to be associated with better QoL, resilience, and self-efficacy, as well as lower mental distress, while the opposite is the case for crisis of meaning (Schnell, 2021; Sørensen et al., 2019). According to Knitzek et al. (2021) meaning in life is an underestimated asset for people’s well-being. The experience of meaning in life is particularly relevant in older people, where meaning-making processes may be viewed as ways of transcending and adapting after personal losses and when struggling with despair (Wong, 1989). It has been found that older people score higher on meaningfulness compared to younger ones (Sørensen et al., 2021). However, it is possible that the oldest old experience a lower degree of meaning in life, as shown by Aftab et al. (2019). Further, older people’s meaningfulness is to a greater extent linked to communal and transcendent factors, compared to more individualistic and materialistic elements in younger age groups (Reker and Wong, 2012). At the same time, people in their sixties are the least likely to experience crisis of meaning (Schnell, 2021). However, those over 70 years of age tend to experience more crisis of meaning, explained by a shift in status, not being useful to others, and lacking responsibilities in their lives (Vogel, 2010).

Knowledge of meaning in life among nursing home residents with dementia is scarce. It has been argued that people with dementia need tranquility to be acknowledged as human beings, and that everyday meaningful activities are important to this group (Ødbehr et al., 2015). Health professionals working with people with dementia report dealing with different forms of meaning in the caring situation with this target group (Isene et al., 2021a). It has been emphasized that awareness of meaningfulness and crisis of meaning among nursing home residents with dementia may be of high value when integrated into health professionals’ clinical practice, thus improving dementia care. Dewitte et al. (2019) found in a cross-sectional study that the presence of meaning is related to well-being outcomes in nursing home residents with Alzheimer’s Disease (AD) (Dewitte et al., 2019). The same research group conducted a longitudinal study finding that nursing home residents with AD with a higher presence of meaning had less severe depressive symptoms 1 year later (Dewitte et al., 2022). The same study also questions the prevailing view that the presence of cognitive abilities is necessary for the experience of meaning in life, since no such association was seen in the study (Dewitte et al., 2022).

### Person-centered care and existential needs

To provide high-quality care and good life conditions for people with dementia in nursing homes, person-centered care has emerged as an approach in the care of older people (Terkelsen et al., 2020). Person-centered care aims to focus on nursing home residents’ personhood, autonomy, QoL and well-being, including existential aspects as essential components (Isene et al., 2021a). Meaning in life is essential in connection with such existential aspects in health, since “the existential is expressed primarily through a quest of meaning and seeking meaning in life in general, as well as in demanding life situations” (Nygaard et al., 2022). By mapping the nursing home residents’ meaning in life, generated knowledge may have implications for health professionals’ performance of person-centered care and their choices when supporting residents’ existential resources. Such knowledge may also encourage health professionals to accommodate situations in everyday life settings facilitating meaning-making activities (Isene et al., 2021b). Overall, this kind of mapping and health professionals’ actions based on it may have the potential to improve residents’ QoL and mental health status.

### The need for a suitable tool – developing MIND

For quite some time, the biomedical approach dominated research in dementia. For this reason, research on meaning in life as a positive aspect of psychological functioning has been performed to a limited extent (Shiells et al., 2020). Part of this picture may be the lack of an instrument developed for this purpose in this patient group. Despite growing attention to the role of meaning in life for psychological well-being, few tools have been developed or validated to assess these experiences in individuals with dementia, particularly in nursing home settings. To meet this deficiency, the Meaning in Life in Persons with Dementia Questionnaire (MIND) was developed.

The development of MIND was based on previous empirical work showing a two-dimensionality of meaning in life expressed by the two factors “meaningfulness” and “crisis of meaning” (Schnell, 2021). As such, MIND shares the same foundation as the Meaning and Purpose Scale (MAPS) (Schnell and Danbolt, 2023) and the Sources of Meaning and Meaning in Life Questionnaire (SoMe) (Schnell, 2009, 2011; Sørensen et al., 2019).

Three guiding principles were followed in the development process: (1) validity: MIND should assess meaningfulness and crisis of meaning in a specific target group of nursing home residents with dementia. (2) Feasibility: the items should be adapted to the patient group and suitable for conducting reliable interviews. (3) Acceptability: items should be non-normative and open, to ensure that they did not seem offensive or embarrassing to the target group.

First, to ensure content validity possible items were discussed in a group of researchers with high expertise in geriatric psychiatry in nursing homes and research on existential health and meaning in life. Item suggestions inspired by MAPS and SoMe were compared with items from other instruments used for similar populations, such as the Quality of Life in Late-Stage Dementia scale (QUALID) (Weiner et al., 2000) and the Quality of Life in Alzheimer’s Disease (QoL-AD) (Logsdon et al., 1999). Furthermore, the items were discussed with research nurses trained in conducting similar studies and collecting data among nursing home residents with dementia. As part of this adaptation process, the items were tested in an interview with six people with dementia, followed by a revision of the items. As dementia progresses, the cognitive ability to understand and appreciate an abstract concept is often difficult. Thus, the new statement phrases, and scorings based on MAPS and SoMe, were difficult to understand for some of the people interviewed. They were unable to recognize that a statement presented in the first person by the interviewer was about themselves. Furthermore, a scoring using a combination of numbers on a scale from ‘strongly disagree’ to ‘strongly agree’ was too complicated. In line with these instruments and assessments for the same patient group, the number of items was reduced to as few as possible, statements were changed to questions, and the number of response options was reduced from six to four, to strengthen feasibility. After testing the new version with another 11 participants, the final version of MIND was established.

Additionally, the final six questions were used as an interview guide in a qualitative study of 10 nursing home residents with dementia (Nylund et al., 2025^1^). The main finding was that the residents understood and were able to elaborate on how they perceived the questions and provided narratives about what they associated with meaningfulness and crisis of meaning.

### Purpose of the study

Based on findings from the general population, it could be expected that the experience of meaning in life in this group would be positively associated with QoL and negatively with mental distress. Such knowledge could be important for how person-centered care is performed by facilitating meaning-making activities in everyday life in nursing homes (Isene et al., 2021b). However, there might be other factors interplaying with these associations, such as physical health, cognitive impairment, and demographic factors. On this basis, the aim of the present study was to evaluate the applicability of the newly developed MIND questionnaire among older people with dementia by examining its factor and construct validity, internal consistency, and clinical relevance in a nursing home setting.

## Materials and methods

### Setting and participants

This cross-sectional study employed data from the SAM-AKS study (the Cooperation between the Department of Old Age Psychiatry, Innlandet Hospital Trust, and municipal nursing homes in the Innlandet County) (Vossius et al., 2022; Helvik et al., 2021). SAM-AKS is an ongoing study that started in 2014. Data were collected from the residents at admission to the nursing home and thereafter each year as long as they were admitted to the nursing home. A wide range of health-related parameters relevant to residents in nursing homes, including cognitive function, QoL, mental health, and physical health were collected.

Residents were eligible if they (1) were ≥60 years, (2) had a diagnosis of dementia (any subtype), and (3) had an expected survival of at least 4 weeks after admission. Based on all available information, no cognitive impairment, mild cognitive impairment (MCI), and dementia, as well as dementia subtypes were independently diagnosed by two investigators, both experienced old age psychiatrists and researchers. Disagreements were resolved through consensus discussion with a third senior therapist. Participants diagnosed with dementia were included in the study. The final purposive sample consisted of 116 participants included from 34 different nursing homes in Innlandet County, Norway.

### Data collection

Health professionals at nursing homes received a standardized two-day training session covering study protocols and ethical considerations before collecting data to the SAM-AKS study. As a sub-project in the SAM-AKS study, the MIND data were collected by trained clinical nurses between March 2022 and May 2023. These nurses had received training in the administration of the MIND instrument and ethical considerations. A guide for conducting the interviews was followed.

The present study employed the following measures:

#### Demographic variables

Gender and age.

#### The meaning in life in persons with dementia questionnaire (MIND)

The MIND questionnaire assessed two dimensions of meaning in life, each with three items: ‘Meaningfulness’ (1. “Do you see a meaning in your life?,” 2. “Do you experience your life as meaningful?,” 3. “Do you think your life is worth living?”) and ‘crisis of meaning’ (4. “Does your life seem empty?,” 5. “Do you lack meaning in your life?”). The statements were scored on a four-point scale from “not at all” (0), “a little” (1), “quite a lot” (2), to “a lot” (3). Item 6 (“Does it bother you that you cannot see any meaning in your life?”) was only administered if participants indicated any level of crisis on item 5. If item 5 was rated ‘not at all,’ item 6 was imputed as ‘not at all’. The original Norwegian version of MIND with an English translation is found in Supplementary File 1.

The MIND data was collected as structured interviews by trained nurses to make the questions more accessible for the respondents. Total mean scores were calculated for the two dimensions, ranging from zero to three, where a higher score meant more meaningfulness or more crisis of meaning (Schnell, 2009).

#### The quality of life in Alzheimer’s disease (QoL-AD)

QoL-AD (Logsdon et al., 1999) was used for participants able to self-report (*N* = 52). QoL-AD was measured through 13 items covering different domains such as physical health, mood, living conditions, family, relationships with others, pleasure, self-assessment of own situation, and overall QoL. The items were scored from “poor” (1) to “very good” (4), and the total score of QoL-AD ranges from 13 to 52, with a higher score indicating better QoL. QoL-AD was self-reported through interviews with trained health professionals. QoL-AD has been shown to be appropriate in the Norwegian context (Strandenæs et al., 2022). Cronbach’s alpha for the items of QoL-AD in the present sample was 0.80.

#### Quality of life in late-stage dementia (QUALID)

QUALID was used for those with severe cognitive impairment (*N* = 71), as shown in corresponding samples (Castro-Monteiro et al., 2016). The Norwegian version (Røen et al., 2015) of QUALID (Weiner et al., 2000) was obtained from health professionals’ observation of the participants’ behavior and emotions through 11 items indicating positive and negative dimensions of QoL. The items were rated on a five-point Likert scale with a total score ranging from 11 to 55. A low score indicates a higher level of QoL. Cronbach’s alpha for the 11 items was 0.84 in the present sample.

#### The Cornell scale for depression in dementia (CSDD)

Depressive symptoms were measured by the CSDD (Alexopoulos et al., 1988) through its Norwegian version (Stensvik et al., 2021). The CSDD contains 19 items where each item is rated as “absent” (0), “mild” (1), or “severe” (2). The total score ranged from 0 to 38. A score of 10 or more indicated a probable major depression^2^. The CSDD was rated by health professionals at the nursing homes based on their observations and assessment of the patients’ mental health status. Cronbach’s alpha for the CSDD items in the present sample was 0.82.

#### General medical health rating scale (GMHR)

The GMHR graded physical health as “poor” (1), “fair” (2), “good” (3) and “very good” (4) (Lyketsos et al., 1999). The scores were dichotomized as “poor/moderate” and “good/very good” in the analysis. The GMHR was rated by health professionals at nursing homes, based on their knowledge of the participants.

#### Mini-mental status examination (MMSE)

Cognitive impairment was measured by the Norwegian validated version of MMSE (Engedal et al., 2023). Its normative scores have been presented. The average MMSE score in the sample was 17.59 (SD = 5.44), with scores ranging from 2 to 30. Higher score indicated better cognitive function. The cut-off between normal cognitive functioning and possible cognitive impairment indicating possible dementia is usually set at 23/24.

#### Clinical dementia rating (CDR)

Based on memory, orientation, judgment and problem solving, community affairs, home and hobbies, and personal care, the CDR (Morris, 1997) is a global score grading the severity of cognitive impairment (Eldholm et al., 2018). The CDR is a six-item instrument using an algorithm with emphasis on memory, giving the following scores: no dementia (0), questionable dementia (0.5), mild dementia (1), moderate dementia (2), and severe dementia (3).

### Analysis

We used descriptive statistics to characterize the sample. Internal consistency was expressed by Cronbach’s alpha. The two-factor structure was tested by confirmatory factor analysis (CFA). Bivariate relationships were displayed through Spearman correlations due to a skewed distributed variable. When examining the relationship between the MIND variables and relevant external variables like QoL and depression as measured by QoL-AD, QUALID, and CSDD to demonstrate possible construct validity, we chose to employ multivariate linear regression analysis controlled for possible confounders. Based on an expectation of high covariation between the independent variables CSDD, QUALID, and QoL-AD, we fitted three multiple linear regression models with meaningfulness and crisis of meaning as the dependent variables, respectively, with gender, age, GMHR, and MMSE as control variables. It was checked for deviancy from the normal distribution and multicollinearity. In this way it was possible to examine the independent variables exact predictions on meaningfulness and crisis of meaning.

Due to the exploratory nature of the study, the significance level was set at 0.05.

The analyses were performed on SPSS version 28 and Stata version 18.

### Ethics statement

The present research was approved by the Regional Committees for Medical and Health Research Ethics (2014/917). Informed consent was obtained either from the participants or their next of kin, depending on consent competence. The capacity to provide informed consent for participation in the study was assessed by the health professionals at the nursing homes based on their evaluation of the participants’ understanding, appreciation, reasoning, and ability to express a choice. The interview was interrupted upon signs of resistance or if participants expressed a desire to stop. The purpose of the interview was reiterated several times, and the participants could withdraw from the study at any point.

## Results

### Descriptive statistics and internal consistency

The total mean scores for the two MIND subscales were 1.96 (SD 0.70) for meaningfulness and 0.46 (SD 0.75) for crisis of meaning, respectively (see Table 1). The mean score for CSDD was 4.22, and 13.2% of the participants scored 10 or more on CSDD, indicating clinically significant depressive symptoms. The mean score for QUALID was 18.65 and for QoL-AD 37.44 (see Table 1). The mean score for MMSE was 17.59, and 37.1% of the participants had mild dementia and 36.2% had moderate dementia based on the CDR measurements. Cronbach’s alpha for the items of meaningfulness was 0.86 and for the items of crisis of meaning 0.92, respectively (see Table 1).

**Table 1:** Sample characteristics.

	Values	(SD)	Range	Cronbach’s alpha	*N*
Gender (%)					
Women	64.7				75
Age	85.57	(7.44)	66–101		116
GMHR (%)					
Poor/moderate	45.1				51
Good/very good	54.9				62
CDR (%)					
No dementia	1.7				2
Questionable dementia	14.7				17
Mild dementia	37.1				43
Moderate dementia	36.2				42
Severe dementia	9.5				11
MMSE	17.59	(5.44)	2–30	0.86	110
CSDD^A^	4.22	(4.40)	0–22	0.82	114
QUALID	18.65	(6.56)	11–37	0.84	71
QoL-AD	37.44	(5.80)	21–49	0.80	52
Meaningfulness	1.96	(0.70)	0–3	0.86	104
Crisis of meaning	0.46	(0.75)	0–3	0.92	95

GMHR, General Medical Health Rating scale; CDR, Clinical Dementia Rating; MMSE, Mini Mental Status Examination; CSDD, Cornell Scale for Depression in Dementia; QUALID, Quality of Life in Late-Stage Dementia scale; QoL-AD, Quality of Life in Alzheimer’s Disease.

^A^13.2% of the participants scored 10 or more on CSDD, indicating clinically significant depressive symptoms.

Fifty-two participants in the sample completed the QoL-AD. Among these participants, the mean age was 84.7 years (SD = 5.6), with 61.5% being women, and mean MMSE score was 19.29 (SD = 4.94) (see Table 2). Seventy-one participants in the sample completed the QUALID. Among these participants, the mean age was 86.0 years (SD = 8.01), with 64.8% being women, and the mean MMSE score was 16.08 (SD = 5.13) (see Table 2).

**Table 2:** Age, gender, and MMSE for those completing QoL-Ad and QUALID.

	QoL-AD sample, *N = 52*	QUALID sample, *N = 71*
Age, mean (SD)	84.70 (5.63)	86.00 (8.01)
Women, %	61.5	64.8
MMSE, mean (SD)	19.29 (4.94)	16.08 (5.13)

### Factorial validity (CFA)

When testing the two-factor solution, the CFA displayed that all included goodness of fit-values were clearly on the right side of the limits (see Table 3). The *p* (*x*^2^/df > 0.05) was 0.39 (Moore et al., 2013). Both CFI = 0.999 and Tucker–Lewis Index = 0.998 were higher than 0.95 (Hu and Bentler, 1999). At the same time RMSEA was 0.026 and SRMR 0.03 respectively, both assessed as a good fit (<0.05) (Schermelleh-Engel et al., 2003).

**Table 3:** Goodness of fit indices for two-factor solution of MIND.

	*p* (*x*^2^/df > 0.05)	CFI	Tucker–Lewis Index	RMSEA	SRMR
Two-factor solution	0.39	0.999	0.998	0.026	0.03

**Figure 1:**
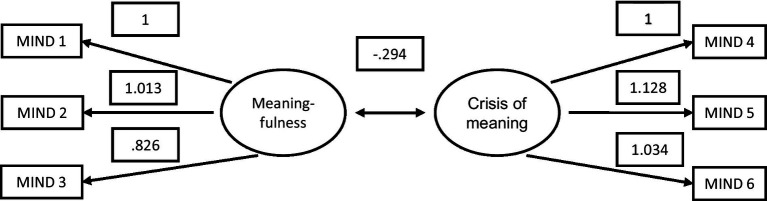
CFA factor loadings (unstandardized).

The CFA factor loadings are presented in Figure 1.

### Construct validity – correlation analyses

There was a highly significant negative correlation between meaningfulness and crisis of meaning (−0.59, *p* < 0.001) (see Table 4). A significant positive correlation was found between meaningfulness and QoL-AD (0.32, *p* < 0.001). Further, a highly significant correlation between CSDD (depressive symptoms) and QUALID (negatively scored) was revealed (−0.74, *p* < 0.001) (see Table 4).

**Table 4:** Correlations between the main independent and dependent variables.

	Meaningful-ness	Crisis of meaning	CSDD	QUALID	QoL-AD
Meaningfulness	1				
Crisis of meaning	−0.59**	1			
CSDD	0.10	−0.03	1		
QUALID^1^	−0.04	−0.02	0.74**	1	
QoL-AD^2^	0.32*	−0.23	−0.15	−0.20	1

*Correlation significant at the 0.05-level, **Correlation significant at the 0.01-level. QUALID is negatively scored. Spearman correlation is employed due to skewed distribution. CSDD, Cornell Scale for Depression in Dementia; QUALID, Quality of Life in Late-Stage Dementia scale; QoL-AD, Quality of Life in Alzheimer’s Disease. ^1^*N* = 71. ^2^*N* = 52.

### Construct validity – regression analyses

In the multivariate linear regression analysis, all models were adjusted for age, sex, MMSE score, and physical health (GMHR). In the regression model predicting meaningfulness, QoL-AD remained a significant positive predictor (standardized *β* 0.346, *p* = 0.023) (see Table 5). CSDD and QUALID were not associated with meaningfulness. A positive and significant relationship between physical health (GMHR) and meaningfulness in the model investigating QUALID and meaningfulness was also observed (standardized *β* 0.343, *p* = 0.029) (see Table 5).

**Table 5:** Multivariate linear regression of the association between CSDD, QUALID, QoL-AD, and meaningfulness (dependent variable), controlled for gender, age, GMHR, and MMSE.

	Unstandardized *β*	Confidence interval	Standardized *β*	*p*-value
Gender	−0.097	−0.595–0.217	−0.065	0.542
Age	−0.001	−0.022–0.020	−0.008	0.939
GMHR	0.166	−0.141–0.473	0.117	0.286
MMSE	0.011	−0.018–0.041	0.082	0.447
CSDD	0.010	−0.024–0.045	0.064	0.548
Gender	−0.068	−0.412–0.276	−0.055	0.693
Age	0.006	−0.016–0.029	0.087	0.568
GMHR	0.407	0.044–0.771	0.343	**0.029**
MMSE	−0.005	−0.043–0.034	−0.033	0.815
QUALID^1^	0.004	−0.025–0.033	0.044	0.771
Gender	0.091	−0.401–0.548	0.055	0.710
Age	−0.016	−0.059–0.028	−0.109	0.469
GMHR	−0.025	−0.531–0.481	−0.015	0.921
MMSE	0.021	−0.029–0.070	0.122	0.407
QoL-AD^2, 3^	0.048	0.007–0.088	0.346	**0.023**

Numbers in bold indicate significance level less than 0.05. GMHR, General Medical Health Rating scale; MMSE, Mini Mental Status Examination; CSDD, Cornell Scale for Depression in Dementia; QUALID, Quality of Life in Late-Stage Dementia scale; QoL-AD, Quality of Life in Alzheimer’s Disease. ^1^*N* = 71. ^2^*N* = 52. ^3^Adjusted *R*^2^ for the model including QoL-AD was 0.021.

In the other regression analysis, none of the included variables were significantly associated with crisis of meaning (see Table 6). However, the standardized β (−0.262) for the association between QoL-AD and crisis of meaning was at the same level as for the association between QoL-AD and meaningfulness (Table 5).

**Table 6:** Multivariate linear regression of the association between CSDD, QUALID, QoL-AD, and crisis of meaning (dependent variable) controlled for gender, age, GMHR, and MMSE.

	Unstandardized *β*	Confidence interval	Standardized *β*	*p*-value
Gender	−0.050	−0.389–0.288	−0.032	0.768
Age	−0.009	−0.033–0.015	−0.083	0.464
GMHR	−0.234	−0.574–0.105	−0.150	0.174
MMSE	0.007	−0.026–0.040	0.045	0.677
CSDD	−0.002	−0.039–0.034	−0.013	0.905
Gender	0.170	−0.188–0.528	0.132	0.344
Age	−0.010	−0.034–0.014	−0.123	0.409
GMHR	−0.283	−0.666–0.100	−0.224	0.144
MMSE	0.007	−0.032–0.045	0.049	0.730
QUALID^1^	−0.008	−0.037–0.022	−0.077	0.600
Gender	−0.459	−1.040 – 0.122	−0.246	0.119
Age	−0.016	−0.071–0.039	−0.090	0.565
GMHR	−0.228	−0.813–0.356	−0.124	0.434
MMSE	−0.007	−0.064–0.051	−0.036	0.814
QoL-AD^2, 3^	−0.040	−0.088–0.007	−0.262	0.094

GMHR, General Medical Health Rating scale; MMSE, Mini Mental Status Examination; CSDD, Cornell Scale for Depression in Dementia; QUALID, Quality of Life in Late-Stage Dementia scale; QoL-AD, Quality of Life in Alzheimer’s Disease. ^1^*N* = 71. ^2^*N* = 52. ^3^Adjusted *R*^2^ for the model including QoL-AD was 0.028.

## Discussion

### Key findings

In the present study we investigated whether MIND with its sub-scales “meaningfulness” and “crisis of meaning” was applicable for use in people with dementia in nursing homes. Both meaningfulness and crisis of meaning had good internal consistency. The negative correlation between meaningfulness and crisis of meaning supports the theoretical distinction between these constructs. Model fit indices indicated excellent fit, supporting the proposed two-factor structure. When construct validity for these dimensions was examined in bivariate correlation and multivariate regression analyses, QoL assessed with the QoL-AD was positively and significantly associated with meaningfulness. A non-significant negative association was found between QoL-AD and crisis of meaning, but with the standardized β at the same level as for the association between QoL-AD and meaningfulness. QoL assessed with QUALID and symptoms of depression assessed with CSDD were not associated with either “meaningfulness” or “crisis of meaning.”

### Interpretation

Internal consistencies for both “meaningfulness” and “crisis of meaning” were within acceptable limits. These findings correspond with previous studies employing similar analysis on SoMe and MAPS for the general population, which was also the case for the CFA (see Table 3) (Schnell and Danbolt, 2023; Sørensen et al., 2019). These findings confirmed the assumption that the six variables measured the different constructs, i.e., “meaningfulness” and “crisis of meaning,” with three items each. This was also supported by the strong negative correlation between the constructs (see Table 4). A qualitative study investigating meaning in life in nursing home residents with dementia found that the participants were able to indicate what was “meaningfulness” and what was “crisis of meaning” in their lives (Nylund et al., 2025^1^). All together, these findings strengthen the impression that the MIND dimensions measure meaningfulness and crisis of meaning among residents with dementia living in nursing homes.

Regarding construct validation of the MIND variables, we would expect meaningfulness to be positively associated with QoL measures and negatively associated with symptoms of depression, and vice versa for crisis of meaning, based on knowledge of the general Norwegian population (Sørensen et al., 2019). Except for the positive association between QoL-AD and “meaningfulness,” we did not find such associations. There may be several reasons why this did not happen for the other variables in question.

When investigating only those who responded to QoL-AD in a separate sample and only those who responded to QUALID in another, it was seen that those in the QoL-sample had less cognitive impairment (MMSE 19.29, higher score = less impairment) compared to those responding on QUALID (MMSE 16.08). The lack of association between meaningfulness and QUALID, and a present positive association between meaningfulness and QoL-AD, may indicate that MIND functions better among those with less cognitive impairment.

Our sample had mild depressive symptoms (mean CSDD score 4.22), with only 13.2% of the participants scoring 10 or more on CSDD, indicating clinically significant depressive symptoms. This rate is lower than previously reported European estimates (10–52%) (Giebel et al., 2016). The mild depressive symptoms in the sample could explain the lack of a significant association between CSDD and both meaningfulness and crisis of meaning. On the other hand, the level of QoL-AD did correspond with other studies (Beerens et al., 2014; Strandenæs et al., 2022), and an expected positive association with meaningfulness was found. The relationship between QoL-AD and crisis of meaning was not found to be significant. However, the standardized β coefficient was at the same level as for the relationship between QoL-AD and meaningfulness with a corresponding level of adjusted *R* square (see Tables 5, 6). This may indicate a lack of statistical power in the analyses.

It was also possible to identify a pattern as to which variables were significantly related to meaningfulness and crisis of meaning and which were not. Symptoms of depression measured by CSDD and QoL measured by QUALID were both scored by interviewing the health professionals in the nursing homes, while the MIND variables and QoL-AD were scored by interviewing the residents through structured interviews. It is known from studies exploring differences in self-report and proxy-report on QoL measures that participants with dementia rate their QoL higher than relatives and health professionals do; this applies to those living at home (Ydstebø et al., 2018) as well as those living in nursing homes (Griffiths et al., 2020). Previous research has also found that such differences were not influenced by age or cognitive impairment (Moyle et al., 2012). In our study it seems that residents with dementia have a more positive view of their life situation in terms of QoL (self-reported QoL-AD) than the view of the health professionals (QUALID). Yet it should be mentioned that in the present sample the participants were assessed by either QoL-AD or QUALID. However, since the MIND variables are also self-reported, this may explain the positive significant association between meaningfulness and QoL-AD. Additionally, QoL-AD and QUALID measure quite different aspects of QoL, since QoL-AD to a large extent emphasis self-report on mood, living conditions, family, relationships with others, pleasure, self-assessment of own situation, while QUALID has a stronger component of health personnel’s observation of behavior and bodily expressions (Logsdon et al., 1999; Weiner et al., 2000).

The two proxy-reported instruments (CSDD and QUALID) were strongly correlated in the present sample, further underlining the difference between self-reported and proxy-reported measurement tools. The strong correlation could also indicate that both CSDD and QUALID quite widely score the health personnel’s impression of the patients’ bodily expressions and that these instruments thus may have overlapping measures (Røen et al., 2015). In future studies using MIND, it may be important to use self-report measures to explore the relationship between meaning in life and mental health and QoL assessments.

In addition to the use of MIND for the purpose of research, we believe that the use of MIND in clinical settings with people with dementia could be important for enhancing person-centered care. Existential and psychosocial issues are often perceived as rather abstract and difficult themes to approach for the nursing home staff (Ødbehr et al., 2014, 2015). MIND could serve as a practical tool for the staff to start a conversation with their patients about existential values and questions. The use of MIND could also serve as an instrument to elicit sources of meaning for the patients (Schnell, 2021) and thus be the base for person-centered actions to enhance meaningfulness. Since we in the current study have included patients with both mild, moderate and severe dementia, and in a former study experienced that the use of MIND was feasible also for patients with severe dementia (Nylund et al., 2025^1^), we believe that the instrument is also clinically valid for this population. Evaluating the meaning of life for individuals with dementia is not solely a scientific issue; it also raises ethical considerations. Even in the face of cognitive decline, the desire to feel valued and to seek meaning remains. Acknowledging and nurturing this need is essential to providing dignified and compassionate care for those with dementia.

### Strengths and limitations

A strength of the present study was the uniqueness of the sample and its possibilities for research on meaning in life among people with dementia in nursing homes. All included goodness of fit-values generated through CFA were clearly on the right side of the limits for a two-factor solution for MIND. Additionally, the study allowed for the inclusion of relevant control variables in multivariate analyses, however limited in number due to the small sample size.

Collecting data among people with dementia is considered resource intensive. Thus, the final sample was smaller than the desired size. The sample size may have constrained the statistical power of the analyses. Also, bivariate and multivariate analyses may have been affected by the different ways of scoring the instruments used, with proxy-based scoring in the CSDD and QUALID, and self-reported scoring in MIND and QoL-AD. Discrepancies may have occurred between patients and personnel regarding the participants’ experience of depression and QoL. Crisis of meaning’s less consistent results compared to meaningfulness, may be part of this issue. On the other hand, there may be additional factors contributing to the other results related to the crisis of meaning, which could be explored in future research.

Communication may be a challenge when interacting with people who have dementia. There will always be a question if the participants understand the questions asked in the questionnaire. On the other hand, a qualitative study related to the present project showed that participants perceived the content of the items even if the dementia was quite severe (Nylund et al., 2025^1^). Thus, the present study employs both qualitative and quantitative approaches, thereby enhancing the acquisition of knowledge and reinforcing the conclusions regarding the applicability of MIND.

It could also be questioned if social desirability bias was present in the sample. However, to minimize the risk of such bias, data were collected by trained health professionals under supervision of experienced research nurses, or by these research nurses themselves.

The issue arises as to whether MIND is culturally specific to the Norwegian context or if it would be applicable in other contexts as well. Regarding the cross-cultural functionality of other scales measuring meaning in life in population-based samples in several countries, like for instance SoMe (Schnell, 2021), there are good reasons to believe that MIND could appear as a valid instrument and be employed outside Norway.

In the present study, meaningfulness and crisis of meaning measured by MIND was investigated in relation to QoL measures and depression. On the other hand, one could argue that meaning in life is connected to factors beyond QoL and mental health (Baumeister, 1991). Though the present study did not have other measures available, further research employing MIND among people with dementia could investigate its relation to other properties like for instance spirituality, social relations, and connectedness with nature and culture, factors designated as sources of meaning (Sørensen et al., 2019).

## Conclusion

The aim of the present study was to evaluate the applicability of the newly developed MIND questionnaire among older people with dementia by examining its factor and construct validity, internal consistency, and clinical relevance in a nursing home setting. Both meaningfulness (*α* 0.86) and crisis of meaning (α 0.92) had good internal consistency. CFA showed that all goodness of fit-values were clearly on the right side of the limits for a two-factor solution for the six items of MIND, coinciding with the presupposed dimensions of meaningfulness and crisis of meaning. When construct validity for these dimensions was examined in bivariate correlations and multivariate regression analyses, QoL assessed with the QoL-AD was positively and significantly associated with meaningfulness, indicating MIND’s applicability among those with less cognitive impairment. A non-significant negative association was found between QoL-AD and crisis of meaning, but with the standardized β and adjusted *R*^2^ at the same level as for the association between QoL-AD and meaningfulness. QoL assessed with QUALID and symptoms of depression assessed with CSDD were not associated with either meaningfulness or crisis of meaning. On this basis, our study indicates that the MIND instrument is applicable for investigations on meaningfulness and crisis of meaning among older people with dementia, meeting the current need for an instrument measuring meaning in life in this target group. MIND’s clinical relevance in nursing home settings is discussed, emphasizing MIND as a relevant tool in assessing existential well-being in nursing home residents with dementia. In the frame of person-centered care, a vulnerable patient group and their need to find meaning despite impairment may be safeguarded.

In further research, MIND may be tested and evaluated in other cultural contexts. Longitudinal studies might investigate the MIND variables’ impact on QoL, mental health and other relevant factors as well as investigating other factors impact on MIND. MIND could also be integrated into care models and tested in intervention studies.

## Data Availability

The dataset generated and analyzed during the current study is not publicly available due to privacy restrictions. Requests to access the datasets should be directed to torgeir.sorensen@vid.no.

## Footnotes

^1^ Nylund S. M., Danbolt L., Feiring I. H., Bergh S., Lichtwarck B., Kirkevold Ø., et al. (2025). Meaning in life for residents with dementia living in long-term nursing homes: an exploratory qualitative interview-based study. Arch. Psychol. Relig. [accepted]

^2^ https://dementiaresearch.org.au/wp-content/uploads/2016/06/CSDD.pdf

## References

[ref1] Aftab A. Lee E. E. Klaus F. Daly R. Wu T.-C. Tu X.. (2019). Meaning in life and its relationship with physical, mental, and cognitive functioning: a study of 1,042 community-dwelling adults across the lifespan. J. Clin. Psychiatry 81:11357. doi: 10.4088/JCP.19m13064, PMID: 31846240 PMC7138140

[ref2] Alexopoulos G. S. Abrams R. C. Young R. C. Shamoian C. A. (1988). Cornell scale for depression in dementia. Biol. Psychiatry 23, 271–284. doi: 10.1016/0006-3223(88)90038-8, PMID: 3337862

[ref3] Baumeister R. F. (1991). Meanings of life. New York, NY: The Guilford Press.

[ref4] Beerens H. C. Sutcliffe C. Renom-Guiteras A. Soto M. E. Suhonen R. Zabalegui A.. (2014). Quality of life and quality of care for people with dementia receiving long term institutional care or professional home care: the European RightTimePlaceCare study. J. Am. Med. Dir. Assoc. 15, 54–61. doi: 10.1016/j.jamda.2013.09.010, PMID: 24220139

[ref5] Borza T. Engedal K. Bergh S. Selbæk G. (2019). Eldre med depresjon – oppfølging over tre år [older people with depression – a three-year follow-up]. Tidsskr. Nor. Laegeforen. 139:968. doi: 10.4045/tidsskr.18.0968 31686490

[ref6] Castro-Monteiro E. Alhayek-Ai M. Diaz-Redondo A. Ayala A. Rodriguez-Blazquez C. Rojo-Perez F.. (2016). Quality of life of institutionalized older adults by dementia severity. Int. Psychogeriatr. 28, 83–92. doi: 10.1017/S1041610215000757, PMID: 26018746

[ref11] Crumbaugh J. C. Maholick L. T. (1964). An experimental study in existentialism: The psychometric approach to Frankl’s concept of noogenic neurosis. J. Clin. Psychol. 20, 200–207. 14138376 10.1002/1097-4679(196404)20:2<200::aid-jclp2270200203>3.0.co;2-u

[ref7] Dewitte L. Hill P. L. Vandenbulcke M. Dezutter J. (2022). The longitudinal relationship between meaning in life, depressive symptoms, life satisfaction, and cognitive functioning for older adults with Alzheimer’s disease. Eur. J. Ageing 19, 1155–1166. doi: 10.1007/s10433-022-00689-z, PMID: 36506678 PMC9729662

[ref8] Dewitte L. Vandenbulcke M. Dezutter J. (2019). Meaning in life matters for older adults with Alzheimer’s disease in residential care: associations with life satisfaction and depressive symptoms. Int. Psychogeriatr. 31, 607–615. doi: 10.1017/S1041610218002338, PMID: 30722804

[ref9] Eldholm R. S. Barca M. L. Persson K. Knapskog A. B. Kersten H. Engedal K.. (2018). Progression of Alzheimer's disease: a longitudinal study in Norwegian memory clinics. J Alzheimer's Dis 61, 1221–1232. doi: 10.3233/JAD-170436, PMID: 29254085

[ref10] Engedal K. Benth J. Š. Gjøra L. Skjellegrind H. K. Nåvik M. Selbæk G. (2023). Normative scores on the Norwegian version of the Mini-mental state examination. J Alzheimer's Dis 92, 831–842. doi: 10.3233/JAD-221068, PMID: 36847004

[ref12] Frankl V. E. (2004). Man's search for meaning: the classic tribute to hope from the holocaust. London: Rider.

[ref13] Giebel C. Sutcliffe C. Verbeek H. Zabalegui A. Soto M. Hallberg I. R.. (2016). Depressive symptomatology and associated factors in dementia in Europe: home care versus long-term care. Int. Psychogeriatr. 28, 621–630. doi: 10.1017/s1041610215002100, PMID: 26652662

[ref14] Gjøra L. Strand B. H. Bergh S. Borza T. Brækhus A. Engedal K.. (2021). Current and future prevalence estimates of mild cognitive impairment, dementia, and its subtypes in a population-based sample of people 70 years and older in Norway: the HUNT study. J Alzheimer's Dis 79, 1213–1226. doi: 10.3233/JAD-201275, PMID: 33427745 PMC7990439

[ref15] Griffiths A. W. Smith S. J. Martin A. Meads D. Kelley R. Surr C. A. (2020). Exploring self-report and proxy-report quality-of-life measures for people living with dementia in care homes. Qual. Life Res. 29, 463–472. doi: 10.1007/s11136-019-02333-3, PMID: 31646416 PMC6994428

[ref16] Helvik A. S. Bergh S. Šaltytė Benth J. Selbaek G. Husebo B. S. Tevik K. (2021). Pain in nursing home residents with dementia and its association to quality of life. Aging Ment. Health 26, 1787–1797. doi: 10.1080/13607863.2021.1947968, PMID: 34251936

[ref17] Hu L. Bentler P. M. (1999). Cutoff criteria for fit indexes in covariance structure analysis: conventional criteria versus new alternatives. Struct. Equ. Model. Multidiscip. J. 6, 1–55. doi: 10.1080/10705519909540118

[ref18] Isene T.-A. Haug S. H. K. Stifoss-Hanssen H. Danbolt L. J. Ødbehr L. S. Thygesen H. (2021a). Meaning in life for patients with severe dementia: a qualitative study of health care professionals’ interpretations. Front. Psychol. 12:701353. doi: 10.3389/fpsyg.2021.701353, PMID: 34539501 PMC8440833

[ref19] Isene T.-A. Thygesen H. Danbolt L. J. Stifoss-Hanssen H. (2021b). Embodied meaning-making in the experiences and behaviours of persons with dementia. Dementia 21, 442–456. doi: 10.1177/14713012211042979 34530634

[ref20] Knitzek B. Alsaker S. Hagen J. Haugan G. Lehmann O. Nilsen M.. (2021). Meaning-making: a underestimated resource for health? A discussion of the value of meaning-making in the conservation and restoration of health and well-being. Encyclopaideia 25, 5–18. doi: 10.6092/issn.1825-8670/11986

[ref21] Logsdon R. G. Gibbons L. E. McCurry S. M. Teri L. (1999). Quality of life in Alzheimer’s disease: patient and caregiver reports. J. Ment. Health Aging 5, 21–32.

[ref22] Lyketsos C. G. Galik E. Steele C. Steinberg M. Rosenblatt A. Warren A.. (1999). The general medical health rating: a bedside global rating of medical comorbidity in patients with dementia. J. Am. Geriatr. Soc. 47, 487–491. doi: 10.1111/j.1532-5415.1999.tb07245.x, PMID: 10203127

[ref23] Martela F. Steger M. F. (2016). The three meanings of meaning in life: distinguishing coherence, purpose, and significance. J. Posit. Psychol. 11, 531–545. doi: 10.1080/17439760.2015.1137623

[ref24] Moore D. S. Notz W. I. Flinger M. A. (2013). The basic practice of statistics. 6th Edn. NY: W. H. Freeman and Company.

[ref25] Morris J. C. (1997). Clinical dementia rating: a reliable and valid diagnostic and staging measure for dementia of the Alzheimer type. Int. Psychogeriatr. 9, 173–176. doi: 10.1017/S1041610297004870, PMID: 9447441

[ref26] Moyle W. Murfield J. E. Griffiths S. G. Venturato L. (2012). Assessing quality of life of older people with dementia: a comparison of quantitative self-report and proxy accounts. J. Adv. Nurs. 68, 2237–2246. doi: 10.1111/j.1365-2648.2011.05912.x, PMID: 22211637

[ref27] Norwegian Government (2017). Meld. St. 15/white paper (2017–2018). “Leve hele livet”. En kvalitetsreform for eldre [“live your whole life”: A quality reform for older people]. Oslo: The Norwegian Ministry of Health and Care Services.

[ref28] Nygaard M. R. Austad A. Sørensen T. Synnes O. McSherry W. (2022). ‘Existential’ in Scandinavian healthcare journals: an analysis of the concept and implications for future research. Religion 13:979. doi: 10.3390/rel13100979

[ref30] Ødbehr L. Kvigne K. Hauge S. Danbolt L. J. (2014). Nurses’ and care workers’ experiences of spiritual needs in residents with dementia in nursing homes: a qualitative study. BMC Nurs. 13, 12. doi: 10.1186/1472-6955-13-12 PMC4011774 24731548

[ref31] Ødbehr L. S. Kvigne K. Hauge S. Danbolt L. J. (2015). Spiritual care to persons with dementia in nursing homes; a qualitative study of nurses and care workers experiences. BMC Nurs. 14:70. doi: 10.1186/s12912-015-0122-6, PMID: 26715914 PMC4693438

[ref32] Olsen C. Pedersen I. Bergland A. Enders-Slegers M.-J. Jøranson N. Calogiuri G.. (2016). Differences in quality of life in home-dwelling persons and nursing home residents with dementia – a cross-sectional study. BMC Geriatr. 16:137. doi: 10.1186/s12877-016-0312-4, PMID: 27400744 PMC4939817

[ref33] Reker G. T. Wong P. T. P. (2012). “Personal meaning in life and psychosocial adaptation in the later years” in The human quest for meaning: theories, research, and applications. ed. Wong P. T. P.. 2nd ed (New York: Routledge), 433–456.

[ref34] Røen I. Selbæk G. Kirkevold Ø. Engedal K. Lerdal A. Bergh S. (2015). The reliability and validity of the Norwegian version of the quality of life in late-stage dementia scale. Dement. Geriatr. Cogn. Disord. 40, 233–242. doi: 10.1159/000437093, PMID: 26227299

[ref35] Røen I. Selbæk G. Kirkevold Ø. Engedal K. Testad I. Bergh S. (2017). Resource use and disease course in dementia – nursing home (REDIC-NH), a longitudinal cohort study; design and patient characteristics at admission to Norwegian nursing homes. BMC Health Serv. Res. 17:365. doi: 10.1186/s12913-017-2289-x, PMID: 28532443 PMC5441072

[ref36] Schermelleh-Engel K. Moosbrugger H. Müller H. (2003). Evaluating the fit of structural equation models: tests of significance and descriptive goodness-of-fit measures. Methods Psychol. Res. Online 8, 23–74.

[ref37] Schnell T. (2009). The sources of meaning and meaning in life questionnaire (SoMe): relations to demographics and well-being. J. Posit. Psychol. 4, 483–499. doi: 10.1080/17439760903271074

[ref38] Schnell T. (2011). Individual differences in meaning-making: considering the variety of sources of meaning, their density and diversity. Personal. Individ. Differ. 51, 667–673. doi: 10.1016/j.paid.2011.06.006

[ref39] Schnell T. (2021). The psychology of meaning in life. London and New York: Routledge.

[ref40] Schnell T. Danbolt L. J. (2023). The meaning and purpose scales (MAPS): development and multi-study validation of short measures of meaningfulness, crisis of meaning, and sources of purpose. BMC Psychol. 11:304. doi: 10.1186/s40359-023-01319-8, PMID: 37789417 PMC10548553

[ref41] Shiells K. Pivodic L. Holmerová I. Van den Block L. (2020). Self-reported needs and experiences of people with dementia living in nursing homes: a scoping review. Aging Ment. Health 24, 1553–1568. doi: 10.1080/13607863.2019.1625303, PMID: 31163987

[ref42] Sørensen T. Hestad K. Grov E. K. (2021). Relationships of sources of meaning and resilience with meaningfulness and satisfaction with life: a population-based study of Norwegians in late adulthood. Front. Psychol. 12:685125. doi: 10.3389/fpsyg.2021.685125, PMID: 34925118 PMC8674485

[ref43] Sørensen T. la Cour P. Danbolt L. J. Stifoss-Hanssen H. Lien L. DeMarinis V.. (2019). The sources of meaning and meaning in life questionnaire in the Norwegian context: relations to mental health, quality of life, and self-efficacy. Int. J. Psychol. Relig. 29, 32–45. doi: 10.1080/10508619.2018.1547614

[ref44] Stensvik G.-T. Helvik A.-S. Nakrem S. Haugan G. (2021). Cornell’s depression for dementia scale: a psychometric study among Norwegian nursing home residents. Arch. Gerontol. Geriatr. 93:104325. doi: 10.1016/j.archger.2020.104325 33383356

[ref45] Strandenæs M. G. Lund A. Engedal K. Kirkevold Ø. Selbæk G. Benth J. S.. (2022). Self-reported quality of life in people with dementia attending a day-care programme in Norway: a 24-month quasi-experimental study. Health Soc. Care Community 30, 1315–1324. doi: 10.1111/hsc.13455 34032347

[ref46] Terkelsen A. S. Petersen J. V. Kristensen H. K. (2020). Mapping empirical experiences of tom Kitwood’s framework of person-centred care for persons with dementia in institutional settings. A scoping review. Scand. J. Caring Sci. 34, 6–22. doi: 10.1111/scs.12709, PMID: 31111522

[ref47] Vogel R. (2010). Lebenssinn in schweren Erkrankungen älterer Menschen [Meaning in life in older people with serious illnesses]. [Doctoral dissertation: Heidelberg University.

[ref48] Vossius C. Bergh S. Selbæk G. Lichtwarck B. Myhre J. (2022). Cause and place of death in Norwegian nursing home residents. Scand. J. Public Health 52, 159–165. doi: 10.1177/14034948221140195, PMID: 36474362

[ref49] Weiner M. F. Martin-Cook K. Svetlik D. A. Saine K. Foster B. Fontaine C. S. (2000). The quality of life in late-stage dementia (QUALID) scale. J. Am. Med. Dir. Assoc. 1, 114–116, PMID: 12818023

[ref50] WHO. (2024). ICD-11: International classification of diseases 11th revision. Available online at: https://icd.who.int/en (Accessed May 25, 2025).

[ref51] Wong P. T. P. (1989). Personal meaning and successful aging. Can. Psychol. 30, 516–525. doi: 10.1037/h0079829

[ref52] Ydstebø A. E. Bergh S. Selbæk G. Benth J. Š. Brønnick K. Vossius C. (2018). Longitudinal changes in quality of life among elderly people with and without dementia. Int. Psychogeriatr. 30, 1607–1618. doi: 10.1017/S1041610218000352, PMID: 29747721

