# Supplementary material for: The validity of the Meaning in Life in Persons with Dementia Questionnaire (MIND)
Source: Front Psychol. 2025 Aug 14;16:1633401. doi: 10.3389/fpsyg.2025.1633401 (PMC12392781; doi:10.3389/fpsyg.2025.1633401)
Supplement: Supplementary file 1 [file Table_1.docx]

**Supplementary file 1**

Presenting the six final items of the MIND questionnaire in Norwegion and in English translation

The statements are scored on a four-point scale from “not at all” (0), “a little” (1), “quite a lot” (2), to “a lot” (3)

|  | **Norwegian version** | **English translation** |
| --- | --- | --- |
|  | ***Meningsfullhet*** | ***Meaningfulness*** |
| 1 | Ser du en mening i livet ditt? | Do you see a meaning in your life? |
| 2 | Opplever du livet ditt som meningsfullt? | Do you experience your life as meaningful? |
| 3 | Synes du livet ditt er verdt leve? | Do you think your life is worth living? |
|  | ***Meningskrise*** | ***Crisis of meaning*** |
| 4 | Synes du livet ditt virker tomt? | Does your life seem empty? |
| 5 | Mangler du mening i livet ditt? | Do you lack meaning in your life? |
| 6 | *Hvis respondenten svarer ‘litt’, ‘ganske mye’, eller*  *‘veldig mye’ på spørsmål 5, så spør:*  Plager det deg at du ikke kan se noen  mening i livet ditt? | *If the respondent answers 'a little', 'quite a lot', or 'a lot'*  *to question 5, then ask:*  Does it bother you that you can’t see any  meaning in your life? |
