# Supplementary material for: The validity of the Meaning in Life in Persons with Dementia Questionnaire (MIND)
Source: Front Psychol. 2025 Aug 14;16:1633401. doi: 10.3389/fpsyg.2025.1633401 (PMC12392781; doi:10.3389/fpsyg.2025.1633401)
Supplement: Supplementary file 2 [file Table_2.docx]

**Supplementary file 2**

*The Meaning in Life in Persons with Dementia Questionnaire (MIND)*

The MIND questionnaire assessed two dimensions of meaning in life, each with three items: ‘Meaningfulness’ (1. “Do you see a meaning in your life?”, 2. “Do you experience your life as meaningful?”, 3. “Do you think your life is worth living?”) and ‘crisis of meaning’ (4. “Does your life seem empty?”, 5. “Do you lack meaning in your life?”). The statements were scored on a four-point scale from “not at all” (0), “a little” (1), “quite a lot” (2), to “a lot” (3). Item 6 (“Does it bother you that you can’t see any meaning in your life?”) was only administered if participants indicated any level of crisis on item 5. If item 5 was rated ‘not at all,’ item 6 was imputed as ‘not at all’. The original Norwegian version of MIND with an English translation is found in Supplementary file 1.

If the participants were unable to score the statements according to the four-part scale (N between 10 and 18, depending on the variable), they were given the option to respond to the statements with “yes”, “no”, i.e. present or not present, or “cannot be evaluated”. These responses were recoded as “not much” in the initial questions. This was done to increase power in the analyses of the study. It was assessed as a conservative approach to code “yes” as “not much” due to uncertainty about how strongly the respondents agreed with the statement “yes”.

Below, the descriptive, bi-variate, and multivariate analyses of the study are performed, including only those who responded to the graded variables (0-3), cf. the first paragraph above. The findings correspond with the results from the complete sample of all cases, i.e. with both the graded and the recoded dichotomized variables.

**Table 1:** Mean (SD) and Cronbach’s alpha for meaningfulness and crisis of meaning

|  | Values | (SD) | range | Cronbach’s alpha | *N* |
| --- | --- | --- | --- | --- | --- |
| Meaningfulness | 2.04 | .70 | 0-3 | .89 | 91 |
| Crisis of meaning | .51 | .79 | 0-3 | .92 | 90 |

**Table 2:** Correlations between the main independent and dependent variables

|  | Meaningful-ness | Crisis of meaning | CSDD | QUALID | QoL-AD |
| --- | --- | --- | --- | --- | --- |
| Meaningfulness | 1 |  |  |  |  |
| Crisis of meaning | **-.70^**^** | 1 |  |  |  |
| CSDD | .14 | .00 | 1 |  |  |
| QUALID | .01 | .03 | **.74^**^** | 1 |  |
| QoL-AD | **.40^**^** | -.25 | -.15 | -.16 | 1 |

Note: * Correlation significant at the .05-level, ** Correlation significant at the .01-level. QUALID is negatively scored. Spearman correlation is employed due to skewed distribution. CSDD = Cornell Scale for Depression in Dementia, QUALID = Quality of Life in Late-Stage Dementia scale, QoL-AD = Quality of Life in Alzheimer’s Disease.

**Table 3:** Multivariate linear regression of the association between CSDD, QUALID, QoL-AD, and meaningfulness (dependent variable), controlled for gender, age, GMHR, and MMSE.

|  | Unstandardized β | Confidence interval | Standardized β | p-value |
| --- | --- | --- | --- | --- |
| Gender | -.097 | -.437 - .243 | -.065 | .572 |
| Age | .008 | -.015 - .031 | .078 | .508 |
| GMHR | .180 | -.046 - .407 | .186 | .117 |
| MMSE | .004 | -.028 - .036 | .028 | .808 |
| CSDD | .018 | -.018 - .054 | .113 | .327 |
|  |  |  |  |  |
| Gender | -.119 | -.471 - .234 | -.100 | .499 |
| Age | .020 | -.003 - .043 | .291 | .083 |
| GMHR | .298 | .044 - .552 | .386 | **.023** |
| MMSE | -.016 | -.055 - .024 | -.123 | .429 |
| QUALID | .007 | -.021 - .035 | .081 | .606 |
|  |  |  |  |  |
| Gender | .153 | -.361 - .668 | .089 | .549 |
| Age | -.025 | -.074 - .023 | -.162 | .298 |
| GMHR | -.048 | -.401 - .305 | -.042 | .784 |
| MMSE | .009 | -.042 - .060 | .053 | .725 |
| QoL-AD^1^ | .072 | .025 - .119 | .480 | **.004** |

Note: GMHR = General Medical Health Rating scale, MMSE = Mini Mental Status Examination, CSDD = Cornell Scale for Depression in Dementia, QUALID = Quality of Life in Late-Stage Dementia scale, QoL-AD = Quality of Life in Alzheimer’s Disease. ^1^Adjusted R^2^ for the model including QoL-AD was .132.

**Table 5:** Multivariate linear regression of the association between CSDD, QUALID, QoL-AD, and crisis of meaning (dependent variable) controlled for gender, age, GMHR, and MMSE.

|  | Unstandardized β | Confidence  interval | Standardized β | p-value |
| --- | --- | --- | --- | --- |
| Gender | -.017 | -.401 - .366 | -.010 | .928 |
| Age | -.010 | -.037 -.016 | -.092 | .436 |
| GMHR | -.217 | -.477 - .042 | -.196 | .099 |
| MMSE | .001 | -.035 - .037 | .007 | .951 |
| CSDD | -.002 | -.042 - .038 | -.011 | .924 |
|  |  |  |  |  |
| Gender | .172 | -.224 - .569 | .127 | .387 |
| Age | -.010 | -.037 - .016 | -.122 | .446 |
| GMHR | -.249 | -.545 - .047 | -.274 | .098 |
| MMSE | .005 | -.036 - .046 | .036 | .807 |
| QUALID | -.007 | -.039 - .026 | -.062 | .684 |
|  |  |  |  |  |
| Gender | -.454 | -1.101 - .194 | -.231 | .163 |
| Age | -.017 | -.082 - .048 | -.086 | .603 |
| GMHR | -.127 | -.538 - .284 | -.103 | .533 |
| MMSE | -.024 | -.089 - .042 | -.119 | .469 |
| QoL-AD^1^ | -.039 | -.090 - .012 | -.252 | .133 |

Note: GMHR = General Medical Health Rating scale, MMSE = Mini Mental Status Examination, CSDD = Cornell Scale for Depression in Dementia, QUALID = Quality of Life in Late-Stage Dementia scale, QoL-AD = Quality of Life in Alzheimer’s Disease. ^1^Adjusted R^2^ for the model including QoL-AD was .032.
